# Complete genome sequence of *Bacillus subtilis* bacteriophage Adastra

**DOI:** 10.1128/mra.00942-24

**Published:** 2024-12-23

**Authors:** Andrew F. Herbig, Eliana M. Pendergrass

**Affiliations:** 1 Department of Biology, Washburn University, Topeka, Kansas, USA; Loyola University Chicago, Chicago, Illinois, USA

**Keywords:** bacteriophages, *Bacillus subtilis*, genome analysis

## Abstract

Adastra is a lytic bacteriophage that infects *Bacillus subtilis*. Here, we report the sequencing and annotation of the 136,306-bp genome of Adastra and its similarity to other myophages in the SPO1 family.

## ANNOUNCEMENT

*Bacillus subtilis* is a Gram-positive, endospore-forming rod commonly found in soil ecosystems and, at low levels, in the gastrointestinal tracts of animals (1, 2). Bacteriophages infecting *B. subtilis* can influence the population dynamics and ecological niche of this bacterium. In this report, we describe the sequencing and annotation of the complete genome of Adastra, a lytic bacteriophage that replicates within *B. subtilis*.

**Fig 1:**
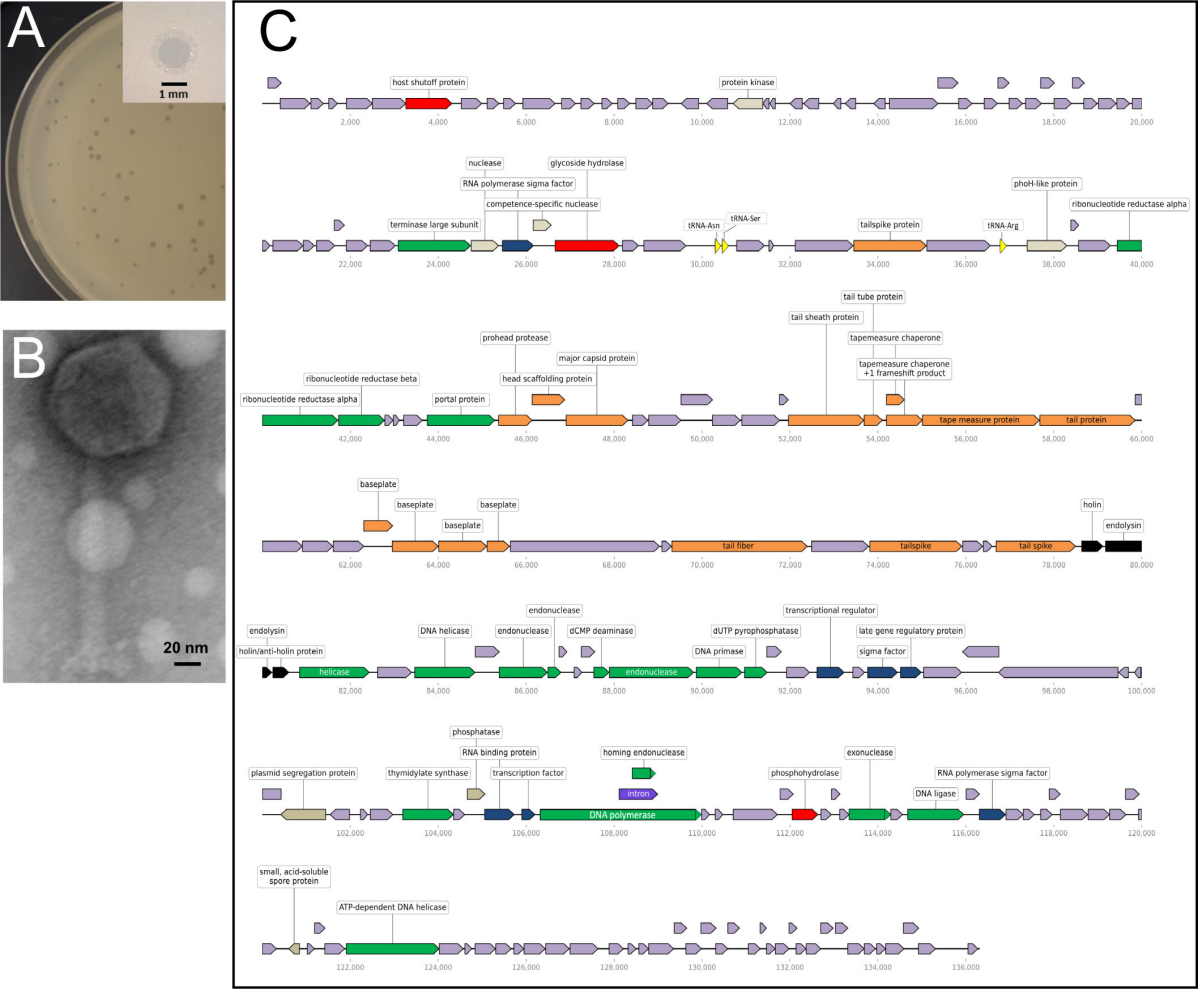
Characteristics of *Bacillus subtilis* phage Adastra. (**A**) The appearance of Adastra plaques on LB agar following infection of *B. subtilis* CU1065 and incubation at 37°C for 18 hours. Inset: low-magnification image of a single plaque. (**B**) Adastra virion morphology. Purified, concentrated phage was absorbed onto 200 mesh copper Formvar/carbon support grids, stained with 2% uranyl acetate, and then imaged with a CM100 (Philips/FEI Company) transmission electron microscope at 100 kV. The Adastra virion has a head width of 73.0 ± 4.4 nm (*n* = 5) and a tail length of 139 ± 3.6 nm (*n* = 5). (**C**) Map of the Adastra genome. The 136,306 bp genome is predicted to have 197 protein-coding genes and three tRNA genes (tRNA-Asn, tRNA-Ser, and tRNA-Arg). Predicted gene product functional categories are indicated by color as follows: Red = host take-over; green = DNA replication and packaging; blue = transcription regulation; orange = phage structure; black = host lysis; yellow = tRNA; purple = intron; gray = miscellaneous functions; lavender = unknown function. Rightward-pointing arrows indicate genes encoded on the top strand while leftward-pointing arrows indicate genes encoded on the bottom strand. Numbers below each row mark the genome scale in base pairs. Map was produced from the Adastra GenBank entry using the Linear Genome Plot tool (v. 1.0) in Galaxy (4).

Adastra was isolated from farmland soil collected near Moline, KS, USA (37°22′03.4″N, 96°19′15.6″W) by incubating 5 g of soil in 50 mL TY Broth (10 g/L NaCl, 5 g/L yeast extract, and 5 g/L peptone) at 37°C with shaking for 24 hours. This mixture was centrifuged at 8,000 rpm for 30 minutes to remove solid material, and the supernatant passed through a 0.22-μm-pore filter. A 500 µL mid-log culture of *B. subtilis* strain CU1065 (3) was infected with 50 µL of filtrate and plaques were detected after incubation at 37°C for 18 hours on LB agar using the double-layer overlay method. A single plaque was picked and purified following three subsequent infections and platings. Purified Adastra produced clear, round, ~1 mm diameter plaques with slightly turbid margins (Fig. 1A). Transmission electron microscopy of Adastra indicated its morphology is similar to SPO1-like phages (Fig. 1B).

Adastra lysate was treated with DNase I prior to genomic DNA isolation with a Norgen Biotek Phage DNA Isolation Kit. Sequencing was performed at the Texas A&M (College Station, TX) Center for Phage Technology (CPT). A DNA library was prepared using an Illumina Nextera library preparation kit and sequenced using a MiSeq V3 2 × 300 bp instrument which generated 300 bp paired-end reads. A total of 94,672 reads were quality controlled with FastQC v.0.11.9 (https://www.bioinformatics.babraham.ac.uk/projects/fastqc/) and trimmed with FASTX-Toolkit v.0.0.14 (5). *De novo* assembly was accomplished with SPAdes v.3.12.0 (6) using default settings which generated a contig with 75.5× coverage. Genes were identified using MetaGeneAnnotator v1.0, GLIMMER v3.0, and ARAGORN v.2.36 (7–9). Transcription terminators were predicted with TransTermHP v.19.1 (10). Gene products were predicted using InterProScan v5.48 (11, 12), BLAST searches against the NCBI nonredundant and Swiss-Prot databases (13), and TMHMM v2.0 (14–16), with default settings for each. All genome sequence assembly workflows and analysis tools were accessed through the CPT Galaxy and Web Apollo interfaces (4, 17, 18). Final annotation for GenBank submission was performed using Artemis v.18.2.0 (19).

The complete Adastra genome is 136,306 bp with 197 predicted protein-coding genes and 3 tRNA genes (Fig. 1C). Only 55 (28%) protein-coding genes have a predicted function. The overall G + C content of the genome is 41% which is comparable to that of its bacterial host. The Adastra nucleotide sequence is 93.5% and 77.6% identical to those of *B. subtilis* myophages SP8 and SPO1 (20, 21), respectively, as determined by EMBOSS Stretcher nucleotide alignments (https://www.bioinformatics.nl/cgi-bin/emboss/stretcher). Direct terminal repeats (DTRs) of 13,597 bp were determined by Sanger sequencing and primer walking from the ends of a region with sequence identity to the DTRs of SPO1 and CampHawk (22) phage genomes (85% and 86% BLASTn nucleotide identity, respectively).

## Data Availability

The genome sequence of phage Adastra has GenBank accession no. PP819608 and BioSample accession no. SAMN41152340. The BioProject accession number is PRJNA1106840, and the SRA accession number is SRR28968994.
